# Exploring Text Mining for Recent Consumer and Sensory Studies about Alternative Proteins

**DOI:** 10.3390/foods10112537

**Published:** 2021-10-21

**Authors:** Ziyang Chen, Cristhiam Gurdian, Chetan Sharma, Witoon Prinyawiwatkul, Damir D. Torrico

**Affiliations:** 1 Centre of Excellence—Food for Future Consumers, Department of Wine, Food and Molecular Biosciences, Faculty of Agriculture and Life Sciences, Lincoln University, Lincoln 7647, New Zealand; Ziyang.Chen@lincolnuni.ac.nz (Z.C.); Chetan.Sharma@lincoln.ac.nz (C.S.); 2 Agricultural Center, School of Nutrition and Food Sciences, Louisiana State University, Baton Rouge, LA 70803, USA; cgurdi3@lsu.edu (C.G.); wprinya@lsu.edu (W.P.)

**Keywords:** alternative proteins, text mining, natural language processing, sentiment analysis

## Abstract

Increased meat consumption has been associated with the overuse of fresh water, underground water contamination, land degradation, and negative animal welfare. To mitigate these problems, replacing animal meat products with alternatives such as plant-, insect-, algae-, or yeast-fermented-based proteins, and/or cultured meat, is a viable strategy. Nowadays, there is a vast amount of information regarding consumers’ perceptions of alternative proteins in scientific outlets. Sorting and arranging this information can be time-consuming. To overcome this drawback, text mining and Natural Language Processing (NLP) are introduced as novel approaches to obtain sensory data and rapidly identify current consumer trends. In this study, the application of text mining and NLP in gathering information about alternative proteins was explored by analyzing key descriptive words and sentiments from *n* = 20 academic papers. From 2018 to 2021, insect- and plant-based proteins were the centers of alternative proteins research as these were the most popular topics in current studies. Pea has become the most common source for plant-based protein applications, while spirulina is the most popular algae-based protein. The emotional profile analysis showed that there was no significant association between emotions and protein categories. Our work showed that applying text mining and NLP could be useful to identify research trends in recent sensory studies. This technique can rapidly obtain and analyze a large amount of data, thus overcoming the time-consuming drawback of traditional sensory techniques.

## 1. Introduction

Several environmental problems have been associated with the rapid increase in meat consumption and related industries. These problems include increased greenhouse gas emissions, nitrates leaching, land compaction, over-consumption of water, and antimicrobial resistance [1,2,3,4]. Thus, to meet the increasing demand for high-quality protein sources in a more environmentally friendly manner, replacing traditional meat with alternative proteins is a potential solution. Currently, there are five main approaches to alternative proteins including plant-based, insect-based, algae-related, fermented by yeast, and cultured meat (or in vitro meat) [5]. Many companies have started to explore the possibility of replacing animal meat-based products with these five types of alternative proteins [1]. To increase the likelihood of successfully commercializing novel products, sensory evaluation plays an important role in product development to optimize foods according to the feedback obtained from consumers [6].

As a key part of sensory science, the development of lexica through traditional approaches requires a large amount of effort, resources, time, and budget, which may sometimes raise barriers and hinder research and development [7]. Simultaneously, the increasing use of web-based platforms to gather information about consumers generates a massive amount of data (so-called big data), which could be of specific interest for fast-moving food companies to identify newer trends, niches, or advantages over competitors. In response to the aforementioned constraints and opportunities, many newer methods, especially those based on advanced computation and artificial intelligence, are paving the way for the development of rapid, efficient, and accurate techniques of data processing. One such technique is text mining, which helps evaluate big data to find meaningful relationships and assertions that would otherwise remain buried in the mass of textual content [8,9]. Analyses of words, sentences, paragraphs, or articles can offer hidden insights that might not be possible to obtain from questionnaires or surveys. Data that can be classified as text are obtained from different sources, including the internet, social media, and scientific reports. However, due to their characteristics and high freedom of word choices, the unprocessed texts tend to be harder to analyze and more time consuming [9,10]. The analyzed text matrix may lead up to thousands of words, and one word may have different meanings in different sentences. To structure this type of analysis, a text’s basic workflow is followed by text segmentation (the process of dividing the main document into smaller parts that are called segments), sentence tokenization (the process of turning sentences into a string of characters called tokens), lemmatization (the process of clustering words and removing inflectional endings), and stemming (the process of removing the suffix from words, which reduces them to root words) [9,10].

All these analyses are barely possible to be finished through manual operation; thus, under this situation, an automatic approach (algorithms) shows significant advantages regarding the optimization of time. Recently, text mining and Natural Language Processing were introduced to help researchers obtain sensory data easier and faster from the internet instead of using repeated sensory tests [11,12,13,14]. This technique can obtain information from different sources (i.e., websites, journals, magazines with consumers’ information), which creates a vast dataset of descriptive words. In general, the obtained lexica from these data mining techniques tend to be “consumer-based” in structure. However, this automation can decrease the time and money spent on research. In addition, it can read a significant amount of sensory data and reform that information into a structured and justified form that is suitable for further analyses. With this technique, sensory research can be conducted more efficiently at the early steps of product development.

For the past few decades, to save time and money in descriptive and consumer analyses, researchers have developed several types of rapid technique. However, all these methods have several shortcomings compared to traditional tests on various levels. The limitation of human processing data has been eliminated with the use of automated algorithms to analyze descriptive data. This research aimed to use text mining and Natural Language Processing to explore structures and meanings about alternative proteins based on the text data collected from scientific reports (*n* = 20 research papers). This research represents a prototype for text mining applications on identifying future food science trends and associations.

## 2. Materials and Methods

### 2.1. Selection of Papers

To obtain the data, one of the most important things is that it is accessible. All of these 20 papers (Table S1) were accessible for hypertext markup language (HTML) and in portable document format (.pdf), which means that they could be scraped by web crawler as well as .pdf text mining commands in R (Version 1.3.1093, Free Software Foundation, Boston, MA, USA) [15] after downloading. Thus, an alternative approach could be developed if the first scraping method did not work. To obtain meaningful insights into the current trends and consumer perception of alternative protein, the criteria for the selection of the scientific papers in this study considered only recently published articles (between 2018 and 2021). Papers’ selection was based on the keywords “alternative protein”, “plant-based”, “insect-based”, “algae-based”, “yeast”, and “cultured meat”. Because they are recent studies, they can provide the latest information and trends of alternative proteins.

### 2.2. Processing of Papers and Texts

All the work was performed in the statistical computing language R (Version 1.3.1093) [15]. The packages applied in R were *rvest* and *xml2* (for web scraping), *pdftool* (for PDF document scraping), *tm* (for text mining), *SnowballC* (for text stemming), *RColorBrewer* (for coloring bar chat and word cloud), *syuzhet* (for emotion analysis and classification), *ggplot2* (for plotting charts) and *wordcloud* (for developing word cloud). Some results were exported as pictures by taking screenshots in portable network graphics (.png) document type in order to improve the pixel of the image.

### 2.3. Text Mining

#### 2.3.1. Web Scraping

**Figure 1:**
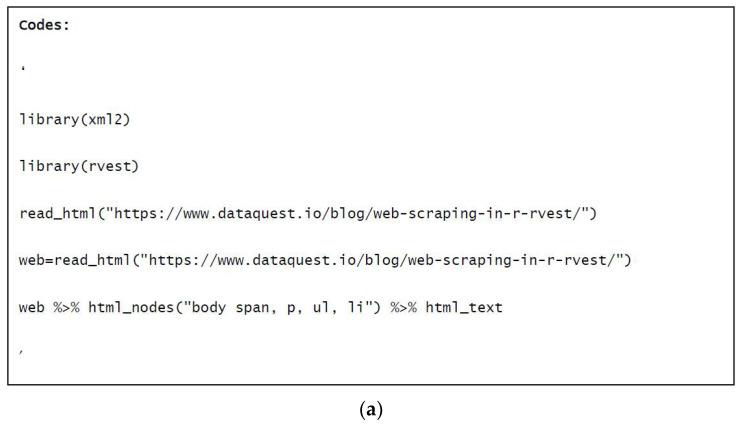

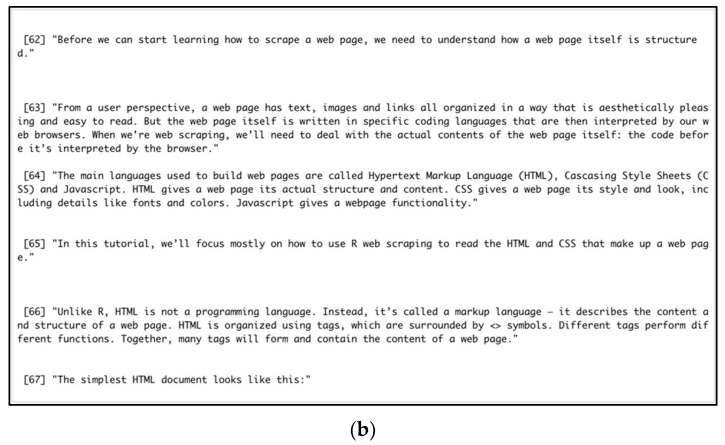
Code (**a**) and a part of the text captured from the website (**b**) by the crawler.

Although grabbing information from a website manually is feasible in some cases [14,16], applying a web crawler would be more advantageous because it saves time. In this case, all the data were collected from scientific reports, which were formatted in PDF. The below figure (Figure 1a) is an example of a simple web crawler performed on a single page (website) to illustrate the basic steps behind web scraping.

The first step was to load the packages which supported the web scraping. In this case, *xml2* (R code) and *rvest* were loaded in the first and second lines, respectively. By applying the “*read_html()*” command and typing the URL into the brackets, this page’s source file was captured in the third line. After this, the Cascading Style Sheets (CSS) information (in the .html document) was used to locate the text, which was needed to be scraped from the page. Normally, these elements of the website could be reached by opening the developing tool in the browser. Finally, by typing the CSS information into the brackets in the “*html_nodes()*” command, all of the text from this webpage was scraped and illustrated in the R console. An example of the scraped information is showed in Figure 1b.

#### 2.3.2. PDF Scraping and Text Processing

**Figure 2:**
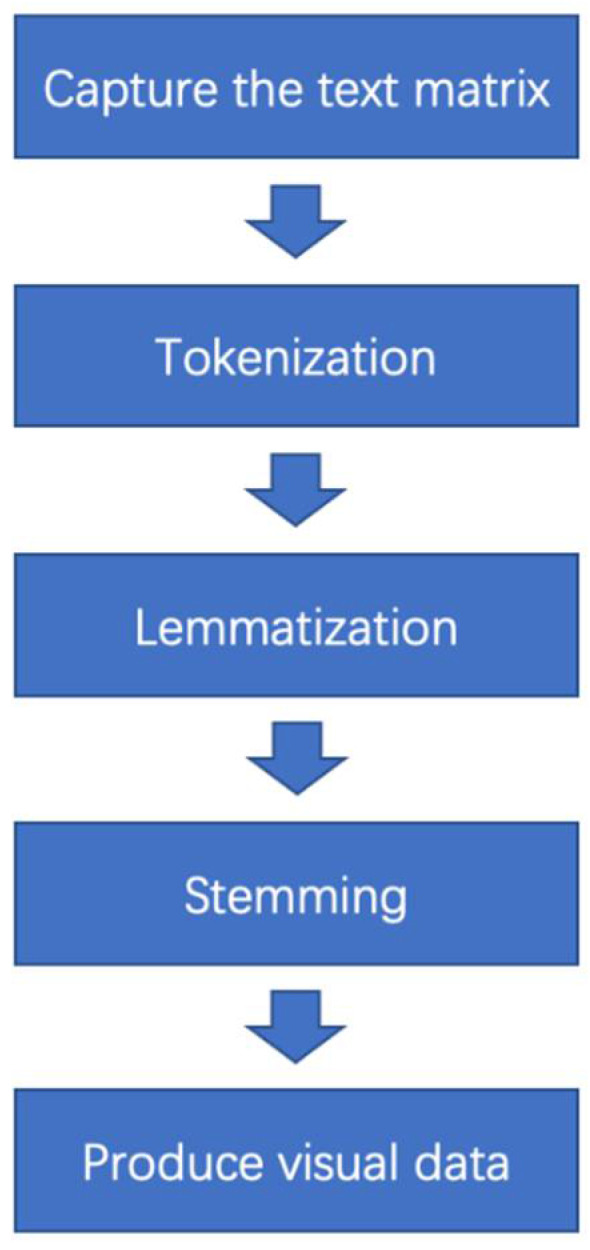
The basic workflow of Natural Language Processing.

Instead of websites, actual .pdf documents were used to scrape the information in this study. The .pdf document scrapping process was similar to the one used for web scraping. The codes applied in this study are shown in Supplementary File S1 and were written by Cristhiam Gurdian from Louisiana State University, USA. The first step was to download the academic articles that were suitable for the research topic. As detailed in Supplementary File S1, the codes required that the working directory was set to the folder containing the PDF files. After the directory was set, the codes were run for the Natural Language Processing (NLP) (Figure 2. text segmentation, sentence tokenization, lemmatization, and stemming). When this step was complete, the text matrix was ready to be analyzed. Word count and other data visualization techniques were produced by applying packages in the R program such as *syuzhet*, *ggplot2*, and word cloud. Additionally, these codes were used to count the keywords in the texts. A more detailed explanation of the procedure and specific codes used to analyze and process the data are shown in Supplementary File S1.

#### 2.3.3. Text Scraping and Natural Language Processing

To obtain more specific data regarding the sensory characteristics of alternative proteins, the objects of analysis in this study were the texts containing the findings from the selected academic papers. The introduction, materials and methods, conclusion, and references sections were excluded, and only the results and discussions parts were extracted for further analysis. The text from academic papers was copied and pasted into a text (.txt) document. There were *n* = 20 analyzed papers, and each result and discussion section of the papers was individually pasted into a new .txt document. After that, the vector containing all the .txt documents were combined to produce a .txt matrix, which was the main object of analysis in this study. Thus, 20 .txt documents that contain the texts from 20 academic papers and one .txt document named “Main Text Matrix” that contained all of the texts from the 20 .txt documents were generated. In total, 21 .txt documents were analyzed. The “Main Text Matrix” was produced to investigate the whole picture of these twenty academic papers regarding the sensory attributes of alternative proteins. All these documents were captured and processed using Natural Language Processing text segmentation, sentence tokenization, lemmatization, and stemming by running the respective codes (shown in Supplementary File S2) before producing any data visualization outputs.

The frequencies of each word occurring in the “Main Text Matrix” were counted and showed in a table and bar chart. In this manner, a preliminary relationship between words and alternative proteins was developed. Sentiment analysis and emotion classification was performed using a package called *syuzhet* (R code) [17]. The frequency of sentiments was counted and the proportion of each emotion in the matrix was illustrated in a bar chart. The emotion classification of the 20 .txt documents was run individually to obtain the proportion of emotional data in each paper. The types of alternative proteins mentioned in each article were also indicated; thus, the emotions associated with each type of alternative protein were explored. A word cloud was produced during the analysis to provide an intuitive image of the frequency of words in the matrix. Based on the word frequency results, the association between words was investigated. This process can show the vocabularies around the terms which were aimed at, as well as the strength of their relationship. More specific and reliable details regarding alternative proteins can be collected by following the word association data.

### 2.4. Statistical Analysis

To obtain the visual relationship between emotions and the types of alternative proteins, the correspondence analysis test was conducted using the XLSTAT software (Version 2018.1.1.62926, Addinsoft Inc., New York, NY, USA) in Excel with a *p* < 0.05 threshold for statistical significance.

## 3. Results and Discussion

**Figure 3:**
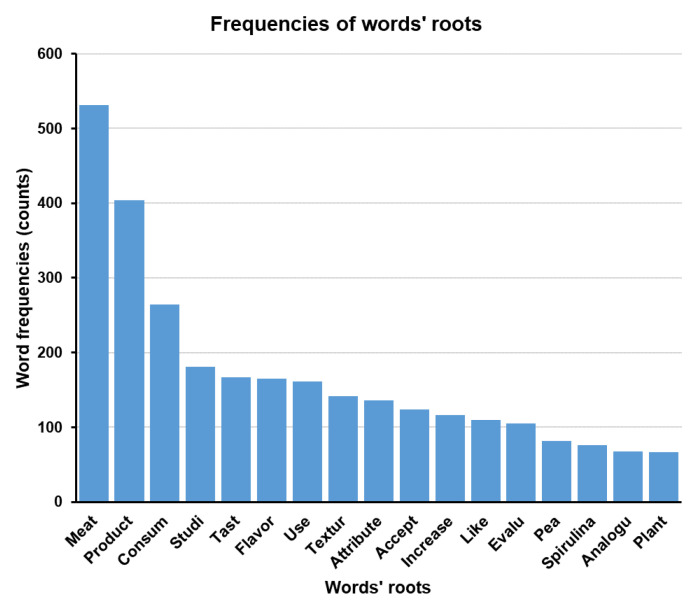
Example of a bar chart showing the frequency of keywords (roots) in the text matrix (*n* = 20 published papers).

**Figure 4:**
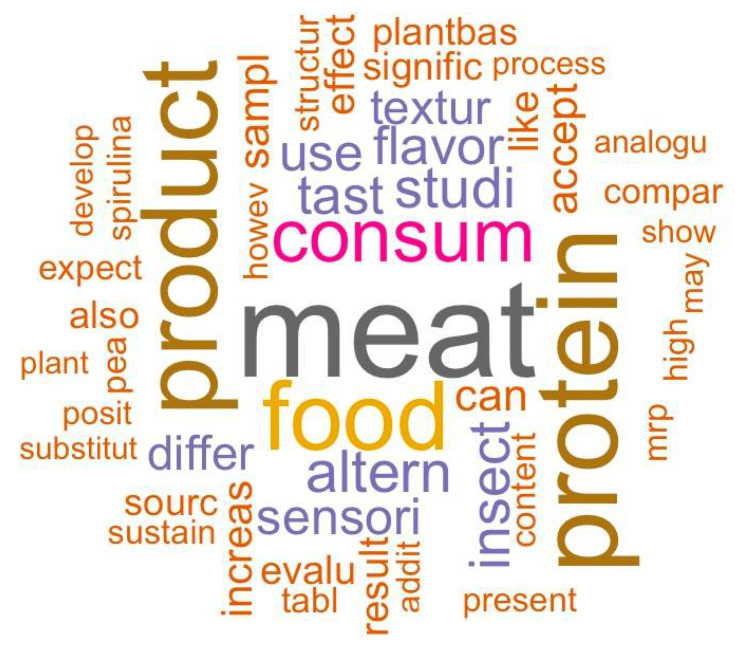
Word cloud obtained of the text matrix (*n* = 20 published papers).

**Figure 5:**
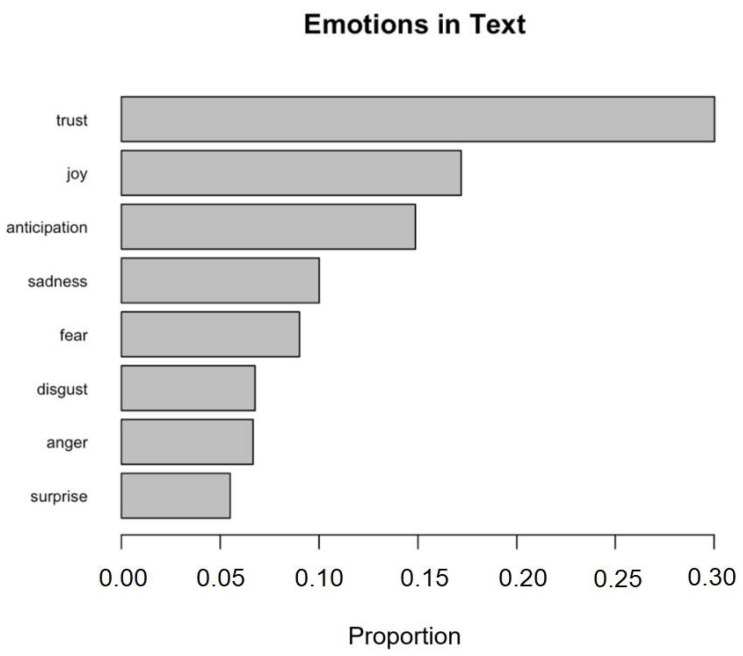
Proportions of emotion terms mentioned in the text matrix (*n* = 20 published papers).

The word frequency results from the “Main Text Matrix” are shown in Figure 3. The detailed word frequency data are shown in Table S2. A word cloud was generated to show the word frequency more intuitively (Figure 4). In the word cloud, the most frequent word appears in the center and the words with higher frequency appear with bigger font size, while the words with lower frequency appear with smaller font size. The proportion of each emotion in the text matrix is indicated in Figure 5. Partial results from the relevance analysis between keywords and other words are shown in Table 1. All the associations between words in the text mining analysis are shown in Supplementary File S3. The proportion of emotions in each paper (20 articles in total) were generated and are shown in Table 2. All the words shown in the tables, figures, and Supplementary Files were in their root form. For instance, “*consum*” would represent “*consumer*”, “*consume*”, “*consumes*”, “*consuming*”, *“consumed*”, and “*consumption*”. Thus, when the frequency of “*consum*” was 264 times, it meant that all the words related to this root appeared 264 times in total.

### 3.1. Word Frequency

A high frequency of “*meat*”, “*protein*”, “*product*”, “*food*”, and “*consum*” root words (531, 432, 404, 356, and 264, respectively) was observed.

The words related to a type of alternative protein were *insect* (179 times), *plantbas* (97 times), *pea* (82 times), *spirulina* (76 times), and *plant* (67 times) as indicated by the top 50 frequent words in the matrix (Table S2). Because the word roots *plantbas* and *plant* have a similar meaning, they were summarized together, representing the total words of plant-based alternative proteins. Thus, among the main categories of research focused on alternative proteins, the insect-based was the most common (179 times) followed by the plant-based (164 times). This suggests that insect- and plant-based proteins were the trendiest topics in alternative proteins scientific research. However, the number of academic papers used in this analysis limits these insights. The performance of text mining could be improved by increasing the size of the text matrix [14].

There were no word-roots associated with cultured meat and/or yeast-fermented proteins that were shown in the top 50 frequency word list (Table S2), which means that they were not relevant in the findings of these 20 articles. The insect-based and plant-based proteins were the most frequent topics in this study, while algae-related proteins (76 times) were less explored, and the cultured meat and yeast-fermented proteins were the least explored alternatives in these studies. A potential explanation for this is that cultured meat and yeast-fermented proteins are in an early stage of development and not many findings have been cited in the current research papers analyzed in this study [6]. Interestingly, instead of the word “*soy*”, the word “*pea*” was the only plant-related word that appeared in the top 50 frequency of words list (Table S2). Based on this result, it can be concluded that researchers are shifting their attention to pea in terms of producing plant-based proteins in recent studies (from 2018 to 2021, which was the year range considered for the analyzed papers). Cosson et al. [18], García-Segovia et al. [19], Kaleda et al. [20], Martin et al. [21], Sha and Xiong [22], Stephan et al. [23], and Yuliarti et al. [24] investigated pea as a plant-based meat alternative protein while only two papers investigated soy as an alternative protein. Moreover, according to Cosson et al. [18], pea protein has become more popular in food products as a plant-based alternative protein due to the enhancement of the food systems’ sustainability. The same approach could also be applied to the word root “*spirulina*”. When the applications of algae alternative protein were explored, “*spirulina*” was the most frequent word in research involving algae protein, which has been recently used in food products [25].

Thus, based on the word frequency in the text matrix, key points can be investigated for further analysis. For instance, in this study, it was found that researchers are mainly focused on exploring insect- and plant-based proteins as potential meat-protein alternatives while cultured meat and yeast-fermented proteins have been mentioned less frequently. Interestingly, pea has become the most common source for plant-based protein application in current research, while spirulina was the most popular algae-based alternative protein in the current studies.

The word roots, which may indicate the attributes of alternative proteins such as “*differ*”, “*accept*”, “*increas*” (the root of increase), “*like*”, and “*posit*” (the root of positive), are also illustrated in Table S2 with frequencies of 141, 124, 116, 110, and 73 times, respectively. To analyze these types of words, caution must be taken in the assumptions derived from the word roots’ frequencies because they are counted regardless of their positive or negative implications in the article. Taking the word root “*differ*” as an example in the text, whether it is positive or not, it would be counted as a word root. The proportions of positive and negative differences in the text matrix were unknown. Thus, no assumption could be made regarding how positive or negative traditional meat products were in relationship to alternative proteins. The same rule applies for the word roots “*accept*”, “*increas*”, and “*like*” because they may represent not acceptable, not increased, and not liked, respectively. Although the antonym of these words could be written as unacceptable, decrease, and dislike, respectively, researchers tend to use their own descriptive words, making it possible that the same root words have been used in both positive and negative connotations. Hence, analyzing the relevance between keywords and other words can be used to support the frequencies of the words findings and improve the reliability of the assumptions made based on text mining.

### 3.2. Relevance between Different Words

The word frequency results can show important insights into the text matrix but neither positive nor negative statements can be inferred. In Table 1, partial results from the relevance analysis (proportion of association) between keywords and other words are shown. For the full results of the relevance analysis, Supplementary File S3 can be referred to. It is noticed that the word “*insect*” had high associations with the words “*willing*”, “*neophobia*”, “*cockroach*”, “*disgust*”, “*novel*”, and “*bit*”. The sensory profile and the acceptance of insect-based alternative protein are reflected by these words [26]. Moreover, the words “*willing*” and “*neophobia*” had similar coefficients (0.37–0.38). It could be assumed that insect neophobia can affect the willingness of trying insect-based alternative proteins [27]. Several articles supported this finding. De Koning et al. [28] found that food neophobia affected the willingness to consume insect protein and impacted plant-based proteins. Similarly, food neophobia caused a negative influence on the acceptability of entomophagy and the sensory appeal of insect-based products [29,30,31]. The words “*cockroach*”, “*disgust*”, and “*novel*” also showed high and similar relationships with the word “*insect*”. It can be concluded that “*cockroach*” was a trendy topic regarding insect-based alternative proteins because this word was mentioned in Chow et al. [30] and García-Segovia et al. [19] studies. In terms of the descriptive words, “*disgust*” and “*novel*” had the highest associations (0.33) with the word “*insect*”. Indeed, entomophagy is still considered a novel practice in Western cultures and “disgusting” was a commonly elicited emotion among participants when they were introduced to the concept of entomophagy [28,29]. Furthermore, insect-based bread has been considered disgusting by participants [19], and the disgust emotion has contributed to the rejection of entomophagy to a greater extent than food neophobia [27,30].

**Table 1:** Relevance (proportion of association) between keywords and other words.

**1. Insect**				
Willing (0.38)	Neophobia (0.37)	Cockroach (0.34)	Disgust (0.33)	Novel (0.33)
**2. Accept**				
Don’t (0.45)	Adult (0.31)	Barrier (0.29)	Elder (0.29)	
**3. Like**				
Tomato (0.41)	Spirulinarel (0.30)	Negat (0.29)		
**4. Plantbase**				
Health (0.26)	Insectsbas (0.26)	Asia (0.25)		
**5. Expect**				
Disappoint (0.35)	Novel (0.30)	Reject (0.29)		
**6. Pea**				
Lupin (0.54)	Mushroomi (0.51)	Dusti (0.49)	Green (0.45)	Nut (0.40)
Earthi (0.38)	Bitter (0.34)			

As mentioned above, an assumption based solely on the frequency of words such as “*accept*”, “*like*”, and “*expect*” is not a reliable approach. These three words were surrounded by negative words sharing a high association level: *“Don’t”* (0.45) was related to *“accept”*; *“Negat”* (0.29), which was the word root of “*negative*”, was related to *“like”*, and *“disappoint”* (0.35) and *“reject”* (0.29) were associated with *“expect”*. These results suggest that alternative proteins had still not been accepted/liked/expected in the studies covered for this research (the negative words were not 100% related to the keywords). To better understand which type of alternative proteins have a negative effect on product acceptability, more data need to be considered. Firstly, plant-, insect-, and algae-based proteins were the text matrix’s main objectives based on the result from the words’ frequencies (Table S2). According to the relevance of insects with other words, it is expected that insect protein could negatively affect the product’s acceptability [26]. Furthermore, in the relevance analysis of words, *“spirulinarel”* (the word associated to spirulina, an alga) was found to be related to *“like”*. Finally, there were no negative words shown in the relevance analysis of *“plantbas”*. Hence, it could be expected that insect-based proteins have lower acceptability among consumers, while the plant- and algae-based proteins have higher expected acceptability [32]. In this study, the information on cultured meat and yeast-fermented proteins was limited, which restricted the development of insights or possible inferences regarding its consumer acceptability.

Indeed, a previous study found that insect-based products tend to have lower acceptability among consumers [27] when compared to other products formulated with alternative proteins, or when compared to control formulations without edible insects. In a different study, according to the sensory evaluation results from *n* = 71 participants, plant-based (soy) meat analogues were as acceptable as beef samples regarding visual appearance [1,33]. In another study, the sausage made from wheat and soy isolates was not significantly different compared to the traditional meat sausage in terms of texture liking [1,34]. Other research also showed that there were no significant differences between a plant-based (soy) meat patty and the all-beef patty regarding overall liking [35]. Furthermore, consumers prefer to adopt plant-based alternative proteins rather than insect-based proteins [28,36]. Usually, consumers refuse to eat insects because of food neophobia and feelings of disgust [28]. Although participants’ acceptability might be improved occasionally through education about the nutritional and environmental benefits derived from insect protein consumption, at first glance, consuming insects was considered a disgusting and unadoptable practice most of the time [19,28,29,30,37].

In Table 1, a part of the developed descriptive lexicon for plant-based and pea alternative proteins is presented. The word *“plantbas”* was related to “*health*”, *“insectbas”*, and “*Asia*”. The consumers tended to agree that plant protein was healthier than meat protein, which has been previously documented by other scientific research [1,18,19,21,28]. Many plant-based protein products, such as tofu, were first introduced in Asia [1,28]. This might explain the observed high relevance between the plant-based protein word and the word “*Asia*”. According to Table S2, the plant- and insect-based proteins presented comparable high-frequency counts. Moreover, these words had a high relevance between them (Table 1). Because the plant-based protein high importance, and pea being the only type of plant that was shown in the top 50 words frequency list, the word “*pea*” was also analyzed as a keyword. According to Table 1, “*pea*” was in high relevance with “*mushroom*” (0.51) and “*lupin*” (0.54) because there were two articles in the text matrix that compared pea protein to mushroom and lupin [18,23]. Because of this, it was difficult to judge whether the following descriptive words were related to pea, mushroom, or lupin. However, in the article, pea was described as green, beany, fresh, and grassy, while lupin was evaluated as beany/green, mushroom/earthy, nutty, and other descriptors [18]. In other words, the dusty and earthy were not related to pea protein [18].

Based on the relevance analysis, it was also found that the acceptability of alternative proteins was related to age. The words “*adults*” (65–70 years old) (association level = 0.31) and “*elder*” (70 or above years old) (association level = 0.29) were highly associated with the word “*like*” in the context of alternative proteins [31]. Food neophobia may be mitigated as age increases [28,29,30,31]. All of these findings agree with the results from the word frequency and relevance analyses from the text mining approach, which were essential for making inferences about the alternative protein topic in this study.

### 3.3. Emotions Analysis

**Figure 6:**
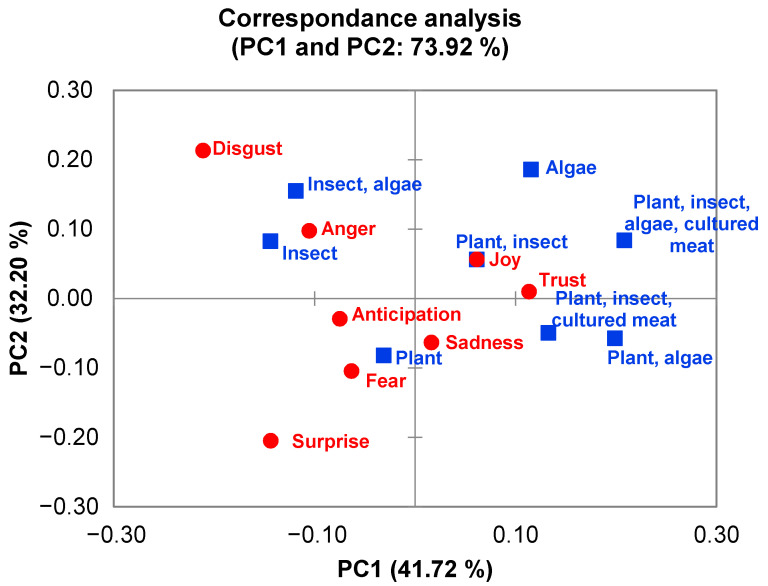
The symmetric plot of correspondence analysis.

The outcome from the analysis of the emotions for the whole text matrix is illustrated in Figure 5. Overall, there was no significant association between the protein types and the emotions (*p* = 0.41) because the observed value for the Chi-square statistic was lower than the established critical value (Chi-square observed = 50.73, and Chi-square critical value = 66.34; Degree of Freedom = 49). Hence, the null hypothesis of independence between the rows and the columns of the contingency table (protein type × emotions) was not rejected. The results from the analysis of emotions present in each paper and the correspondence analysis symmetric plot are shown in Table 2 and Figure 6, respectively.

**Table 2:** Emotion analysis (percentage of representing the emotion) of each academic paper (details can be found in Table S1).

**Paper No.**	**Alternative Protein Types**	**Percentage of Each Emotion in the Text (%)**							
		**Trust**	**Joy**	**Anticipation**	**Sadness**	**Fear**	**Disgust**	**Anger**	**Surprise**
1	Plant	30	18	14	8	10	7	7	6
2	Plant, insect	34	17	16	9	7.5	7.5	9	0
3	Plant, insect	23	14	17	15	11	6	7	7
4	Plant	22	13.5	17	15	11	6.5	7.5	7.5
5	Plant, insect	40	21	13	6.5	5.5	4	5	5
6	Insect, algae	27.5	17.5	19	8	5	11	6	6
7	Plant, insect, cultured meat	32	20	14	11	10	4	4	5
8	Insect	23	16	14	11	11	9.5	11	4.5
9	Plant	35	17	29	4	4	3.5	0	7.5
10	Plant	28	14	16	16.5	13	1	4	7.5
11	Plant	19	14.5	13.5	12.5	13.5	8.5	7.5	11
12	Plant	34	12	26	14	4	4	6	0
13	Plant, algae	41	16	8.5	10.5	7	5	4	8
14	Plant	26	14	13	12	5.5	12.5	10	7
15	Algae	32	23	14	10	5	8	5	3
16	Plant, insect, algae, cultured meat	36	20	15	10	7	4	6	2
17	Plant	30	15	15.5	11.5	10	5	6.5	6.5
18	Insect	31	16.5	20	9.5	2.5	6	5	9.5
19	Plant	32	27.5	11.5	6	11.5	0	0	11.5
20	Insect	24	16	11	9	14.5	14	8	3.5

“Trust” dominated the sentiments in the text matrix with an observed occurrence of 30% (Figure 5). However, “trust” cannot be classified as either positive or negative as this proportion is showing the cumulative occurrence for all contexts. “Joy” had the second-highest proportion in the text matrix, which might indicate that researchers or consumers felt optimistic for the future of the alternative proteins. In previous research, consumers have well accepted plant-based proteins due to their health benefits and relatively pleasant sensory attributes [1,18,19,21,28]. For the insect-based proteins, although cultural barriers affect their preference in the short term, it is expected that insect-based products will eventually become acceptable for the public because of the frequent exposure over time through advertisement, education, and marketing [19]. Algae-based protein, cultured meat, and yeast-fermented proteins had a competitive advantage over other alternative proteins because they require fewer resources to be produced, such as soil and freshwater [6,38,39]. However, these alternatives might require more expensive technological instruments to produce them. The negative emotions, including “sadness”, “fear”, and “disgust”, represented 11, 9, and 6% of the total emotions, respectively (Figure 5). A more detailed profile of the proportions of emotions for each paper can be observed in Table 2. The negative emotions in the text matrix might be viewed as the potential drawbacks of alternative proteins. For example, the text containing food neophobia may reflect fear of the alternative protein sources regarding their effects on health, and the descriptive word “*disgusting*” may indicate mental associations with other disgusting elements that are evoked when exposed to alternative protein sources.

Figure 6 indicates the relationship between emotions and the categories of alternative proteins (based on the keywords of the *n* = 20 papers studied). In the symmetric plot, “*insect*” and “*insect, algae*” were separated from other categories as well as associated with “disgust” and “anger”. Besides this, there was no other noticeable relationship shown. This result agrees with the Chi-square test of independence.

### 3.4. Comparison with Other Text Mining Works

In Bakhtin et al. [11] research, several documents were analyzed to achieve a better understanding of the core research topics and trends in agriculture and food production. The data were collected from several databases, including media, websites, and organizations’ files. Based on text mining, the authors concluded that using fertilizers and chemical agents in farming were the major issues studied in food security and determined that embryo DNA, gene editing, and CRISPR/Cas9 were becoming the centers of genetic research instead of gene modification, which had been popular for years. They foresaw that edible insects, industrial meat production, and industrial food systems would become the focus of extensive research and remarked the higher relevance between food security and biological hazards, fungicides, and pesticides [11]. This research could be classified as a big data analysis with a robust approach to finding the underlying relationship between the terms embedded in a significant amount of text.

In another study, social media, websites, and databased papers were collected to study the underlying relationship between food safety, dietary pattern characterization, consumer opinion, product development, food knowledge discovery, and food supply chain management by text mining [40]. In total, *n* = 57 papers were analyzed in that study using a similar approach to the one used in our study. Their approach included word frequency, word association analysis, and sentiment analysis. Furthermore, these authors explored the application of other novel text mining techniques such as text classification, text clustering, and topic modeling [40]. However, a major drawback of their research was the absence of data visualization techniques, which provide a better appreciation of the relationship among the studied variables than the descriptive text. Compared to our research, although more papers were investigated in their study, two important text mining techniques, including the association and sentiment analyses, were used [40]. The result of the present study would tend to be more science-based compared to works using other databases; this can be an advantage when looking for reliable information in text mining [40]. This research showed the recent trends in scientific exploration regarding the use of alternative proteins. Nevertheless, a higher proportion of social media and internet data can be also beneficial for a consumer-based lexicon development.

## 4. Conclusions

In conclusion, this study analyzed *n* = 20 scientific reports about alternative proteins to explore the application of text mining in sensory research. According to the word frequency results, the insect- and plant-based alternative proteins were the centers of attention in recent research (2018–2021). Moreover, pea was the most studied plant source rather than soy among all plants. According to the results from the word association analysis, the insect-based protein was related to terms such as “*neophobia*”, “*cockroach*”, “*disgust*”, and “*novel*”, while plant-based protein was associated with “*health*” and “*Asia*”. Furthermore, the insect-based protein contributed the most to the observed negative sentiments in the text matrix. Correspondence analysis showed that there was no evident association between the emotion terms and the alternative protein sources, although these associations may become significant by increasing the dataset or the emotion terms under analysis. Despite this, this research shows the implementation of a useful tool to obtain information rapidly on current trends in food science. Further research is recommended with larger datasets, which can include social media and websites.

## Data Availability

The data presented in this study are available on request from the corresponding author.

## Supplementary Materials

The following are available online at https://www.mdpi.com/2304-8158/10/11/2537/s1, Table S1: The list of scientific reports analyzed by the Natural Language Processing, Table S2: The frequency of words in the text matrix (top 50), Supplementary File S1: PDF document text mining codes and explanation produced by Cristhiam Gurdian, Supplementary File S2: Text (TXT) document mining codes obtained from https://www.red-gate.com/simple-talk/sql/bi/text-mining-and-sentiment-analysis-with-r/ (accessed on 1 September 2021), Supplementary File S3: The relevance (association levels) between keywords and other words.
