# Supplementary material for: Exploring Text Mining for Recent Consumer and Sensory Studies about Alternative Proteins
Source: Foods. 2021 Oct 21;10(11):2537. doi: 10.3390/foods10112537 (PMC8620912; doi:10.3390/foods10112537)
Supplement: Supplementary file 1 [file foods-10-02537-s001.zip › foods-1394240-supplementary.pdf]

Table S1. The list of scientific reports analyzed by the Natural Language Processing

Number	Title	Journal	Authors/year
1	Role of Sensory Evaluation in Consumer Acceptance of Plant-Based Meat Analogs and Meat Extenders: A Scoping Review	*Food*	Fiorentini et al. (2020)
2	Drivers and Inhibitors in the Acceptance of Meat Alternatives: The Case of Plant and Insect-Based Proteins	*Food*	De Koning et al. (2020)
3	Nutritional, Microbial, and Sensory Evaluation of Complementary Foods Made from Blends of Orange-Fleshed Sweet Potato and Edible Insects	*Food*	Agbemafle et al. (2020)
4	Impact of Fermentation and Phytase Treatment of Pea-Oat Protein Blend on Physicochemical, Sensory, and Nutritional Properties of Extruded Meat Analogs	*Food*	Kaleda et al. (2020)
5	Physicochemical Properties and Consumer Acceptance of Bread Enriched with Alternative Proteins	*Food*	García-Segovia et al. (2020)
6	Meat Quality Derived from High Inclusion of a Micro-Alga or Insect Meal as an Alternative Protein Source in Poultry Diets: A Pilot Study	*Food*	Altmann et al. (2018)
7	Consumer perceptions of conventional and alternative protein sources: A mixed-methods approach with meal and product framing	*Appetite*	Possidónio et al. (2021)
8	School children cooking and eating insects as part of a teaching program - Effects of cooking, insect type, tasting order and food neophobia on hedonic response	*Food Quality and Preference*	Chow et al. (2021)
9	Importance of additional information, as a complement to information coming from packaging, to promote meat substitutes: A case study on a sausage based on vegetable protein	*Food Quality and Preference*	Martin et al. (2021)
10	Structuring the meat analogue by using plant-based derived composites	*Journal of Food Engineering*	Yuliarti et al. (2021)

11	Effects of Maillard-reacted beef bone hydrolysate on the physicochemical properties of extruded meat alternatives	*Journal of Food Science*	Chiang et al. (2020)
12	Edible mushroom mycelia of Pleurotus sapidus as novel protein sources in a vegan boiled sausage analog system: functionality and sensory tests in comparison to commercial proteins and meat sausages	*European Food Research and Technology*	Stephan et al. (2018)
13	Towards more sustainable meat alternatives: How technical parameters affect the sensory properties of extrusion products derived from soy and algae	*Cleaner Production*	Grahl et al. (2018)
14	Block protocol for conventional profiling to sensory characterise plant protein isolates	*Food Quality and Preference*	Cosson et al. (2020)
15	Alternative protein sources in Western diets: Food product development and consumer acceptance of spirulina-filled pasta	*Food Quality and Preference*	Grahl et al. (2020)
16	Older Consumers' Readiness to Accept Alternative, More Sustainable Protein Sources in the European Union	*Nutrients*	Grasso et al. (2019)
17	Plant protein-based alternatives of reconstructed meat: Science, technology, and challenges	*Trends in Food Science and Technology*	Sha & Xiong (2020)
18	Product appropriateness, willingness to try and perceived risks of foods containing insect protein powder: A survey of US consumers	*International Journal of Food Science and Technology*	Ardoin et al. (2020)
19	Partial and total replacement of meat by plant-based proteins in chicken sausage: evaluation of mechanical, physico-chemical and sensory characteristics	*Journal of Food Science and Technology*	Kamani et al. (2019)
20	Sensory attributes of edible insects and insect-based foods - Future outlooks for enhancing consumer appeal	*Trends in Food Science and Technology*	Mishyna et al. (2020)

Supplementary File S1. PDF document text mining codes and explanation produced by Cristhiam Gurdian

```
#' ---
#' title:  Text mining trial
#' author:  Cristhiam Gurdian (cgurdi3@lsu.edu)
#' date:   2021-January-06
#' ---

install.packages("pdftools")
library(pdftools)
#set working directory to the folder that contains the pdf files
#remove from the pdf names greek characters or symbols because the lappy function will not work with those

#create a vector of PDF file names using the list.files function.
#The pattern argument says to only grab those files ending with "pdf":
files <- list.files(pattern = "pdf$")#only works if you have your working directory set to the folder where you downloaded the PDF files
files #The "files" vector contains all the PDF file names. We'll use this vector to automate the process of reading in the text of the PDF files.

#The pdftools function for extracting text is pdf_text.
#Using the lapply function, we can apply the pdf_text function to each element in the "files" vector and create an object called "text".

text <- lapply(files, pdf_text) #This creates a list object with three elements, one for each document.
length(text)
#Each element in "text" is a vector that contains the text of the PDF file.
lapply(text, length) #The length of each vector corresponds to the number of pages in the PDF file.

####USING TEXT MINING PACKAGE FOR TEXT ANALYSIS
#First load tm package and then create a corpus, which is a database for text.
#instead of working with the "opinions" text" object we created earlier, we start over.

install.packages("tm")
library(tm)
corp <- Corpus(URISource(files),
               readerControl = list(reader = readPDF))#The Corpus function creates a corpus. The first argument to Corpus is what we want to use to create the corpus.
#In this case, it's the vector of PDF files. To do this, we use the URISource function to indicate that the files vector is a URI (Uniform Resource Identifier) source.
# we're telling the Corpus function that the vector of file names identifies our resources.
#The second argument, readerControl, tells Corpus which reader to use to read in the text from the PDF files (readPDF, a tm function).
#The readerControl argument requires a list of control parameters, one of which is reader, so we enter list(reader = readPDF).
#Finally we save the result to an object called "corp".
```

#Now that we have a corpus, we can create a term-document matrix, (TDM) that stores counts of terms for each document.

#The tm package provides a function to create a TDM called TermDocumentMatrix.

```
library(SnowballC)
text.tdm <- TermDocumentMatrix(corp,
                               control =
list(removePunctuation = TRUE,
stopwords = TRUE,
                                         tolower
= TRUE,
stemming = TRUE,
removeNumbers = TRUE,
                                         bounds
= list(global = c(3, Inf))))
```

#The first argument is our corpus. The second argument is a list of control parameters.

#clean up the corpus before creating the TDM. Remove punctuation, stopwords (eg, the, of, in, etc.), convert text to lower case, stem the words,

#remove numbers, and only count words that appear at least 3 times. We save the result to an object called "text.tdm".

```
inspect(text.tdm[1:10,])#first 10 terms
```

#pdf_text function may preserve the unicode curly-quotes and em-dashes used in the PDF files.

#manually use the removePunctuation function with tm_map, both functions in the tm package.

#removePunctuation function has an argument called ucp that when set to TRUE will look for unicode punctuation.

```
corp <- tm_map(corp, removePunctuation, ucp = TRUE)
```

#re-create the TDM, this time without the removePunctuation = TRUE argument.

```
text.tdm <- TermDocumentMatrix(corp,
                               control =
list(stopwords = TRUE,
                                         tolower
= TRUE,
stemming = TRUE,
removeNumbers = TRUE,
                                         bounds
= list(global = c(3, Inf))))
inspect(text.tdm[1:10,])#first 10 terms
```

#findFreqTerms function to find words that occur at least 100 times:

```
findFreqTerms(text.tdm, lowfreq = 100, highfreq = Inf)
```

#To see the counts of those words we could save the result and use it to subset the TDM.

#we have to use as.matrix to see the print out of the subsetted TDM.

```
ft <- findFreqTerms(text.tdm, lowfreq = 100, highfreq = Inf)
as.matrix(text.tdm[ft,])
```

#To see thews

```
ft.tdm <- as.matrix(text.tdm[ft,])
sort(apply(ft.tdm, 1, sum), decreasing = TRUE)
```

Supplementary File S2. Text (TXT) document mining codes obtained from https://www.red-gate.com/simple-talk/sql/bi/text-mining-and-sentiment-analysis-with-r/

```
library("SnowballC")
library("RColorBrewer")
library("wordcloud")
library("syuzhet")
library("ggplot2")
library("tm")

text <- readLines(file.choose())
TextDoc <- Corpus(VectorSource(text))

toSpace <- content_transformer(function (x , pattern ) gsub(pattern, " ", x))
TextDoc <- tm_map(TextDoc, toSpace, "/")
TextDoc <- tm_map(TextDoc, toSpace, "@")
TextDoc <- tm_map(TextDoc, toSpace, "\\|")

TextDoc <- tm_map(TextDoc, content_transformer(tolower))
TextDoc <- tm_map(TextDoc, removeNumbers)
TextDoc <- tm_map(TextDoc, removeWords, stopwords("english"))
TextDoc <- tm_map(TextDoc, removeWords, c("s", "company", "team"))
TextDoc <- tm_map(TextDoc, removePunctuation)
TextDoc <- tm_map(TextDoc, stripWhitespace)
TextDoc <- tm_map(TextDoc, stemDocument)

TextDoc_dtm <- TermDocumentMatrix(TextDoc)
dtm_m <- as.matrix(TextDoc_dtm)
dtm_v <- sort(rowSums(dtm_m),decreasing=TRUE)
dtm_d <- data.frame(word = names(dtm_v),freq=dtm_v)
head(dtm_d, 50)

barplot(dtm_d[1:50,]$freq, las = 2, names.arg = dtm_d[1:50,]$word,
        col ="lightgreen", main ="Top 50 most frequent words",
        ylab = "Word frequencies")

set.seed(1234)
wordcloud(words = dtm_d$word, freq = dtm_d$freq, min.freq = 5,
          max.words=100, random.order=FALSE, rot.per=0.40,
          colors=brewer.pal(8, "Dark2"))

findAssocs(TextDoc_dtm, terms = c("insect","flavor","like"), corlimit = 0.25)


syuzhet_vector <- get_sentiment(text, method="syuzhet")
head(syuzhet_vector)
summary(syuzhet_vector)

bing_vector <- get_sentiment(text, method="bing")
head(bing_vector)
summary(bing_vector)

afinn_vector <- get_sentiment(text, method="afinn")
head(afinn_vector)
summary(afinn_vector)

d<-get_nrc_sentiment(text)
td<-data.frame(t(d))
td_new <- data.frame(rowSums(td[2:253]))
```

```
names(td_new)[1] <- "count"
td_new <- cbind("sentiment" = rownames(td_new),
td_new)
rownames(td_new) <- NULL
td_new2<-td_new[1:8,]
quickplot(sentiment, data=td_new2, weight=count,
geom="bar", fill=sentiment,
ylab="count")+ggtitle("Survey sentiments")

barplot(
  sort(colSums(prop.table(d[, 1:8]))),
  horiz = TRUE,
  cex.names = 0.7,
  las = 1,
  main = "Emotions in Text", xlab="Percentage"
)
```

Table S2. The frequency of words in the text matrix (top 50)

Word	Frequency	Word	Frequency
Meat	531	Plantbas	97
Protein	432	Also	96
Product	404	Sourc	96
Food	356	Compar	85
Consum	264	Tabl	84
Altern	188	Expect	83
Studi	181	Content	82
Insect	179	Pea	82
Tast	167	Structure	82
Flavor	165	Present	78
Use	161	Sustain	78
Sensori	154	Howev	77
Textur	142	Mrp	77
Differ	141	High	76
Attribute	136	Spirulina	76
Can	128	Posit	73
Accept	124	Addit	73
Sampl	121	Mayb	71
Increase	116	Process	71
Effect	111	Develop	69
Like	110	Show	69
Result	107	Analogu	68
Evalu	105	Plant	67
Signific	100	Subsitut	67

Supplementary File S3. The relevance (association levels) between keywords and other words

```
$meat
     altern     product  substitut   labgrown     barrier       lack        use      addit      skill
       0.62        0.51       0.50       0.44        0.42       0.37       0.36       0.36       0.36
   consumpt     process        red      studi      includ      reduc     analog        one        way
       0.35        0.35       0.34       0.34        0.34       0.34       0.33       0.33       0.33
       diet       clean        can     toward    concentr       meal      graea     bryant       also
       0.33        0.32       0.31       0.31        0.30       0.30       0.30       0.30       0.29
       term    particip        due    benefit environment    address      chang     impact        may
       0.29        0.29       0.29       0.29        0.29       0.29       0.28       0.28       0.28
    contain        help      lower       tofu   animalfre      enjoy      monik       pose       unfa
       0.28        0.28       0.28       0.28        0.28       0.28       0.28       0.28       0.28
     assess         mrp      natur       fish       organ     seitan      frame    attract    perceiv
       0.27        0.27       0.27       0.27        0.27       0.27       0.27       0.27       0.26
     compar      differ    ingredi     replac    behavior      label       hoek     improv     nutrit
       0.26        0.26       0.26       0.26        0.26       0.26       0.26       0.25       0.25
   research     percept   siegrist      white       mimic
       0.25        0.25       0.25       0.25        0.25

$protein
      sourc   animalbas   singlecel     altern       vitro    structur       found       studi   crosslink
       0.60        0.41        0.41       0.40        0.40        0.39        0.38        0.37        0.37
     extent      gluten        dont        use       wheat environment    contain       dairi     various
       0.37        0.36        0.35       0.34        0.34        0.34        0.33        0.33        0.33
    product     without     fibrous    belgium     process       intak  seafoodbas     consist    techniqu
       0.31        0.31        0.31       0.31        0.30        0.30        0.30        0.29        0.29
   although       freez         lin    meatbas       deriv       grasp       salad     student     develop
       0.29        0.29        0.29       0.29        0.29        0.29        0.29        0.29        0.28
   properti       uniqu       major       know         egg      nutrit     sustain         due    research
       0.28        0.28        0.28       0.28        0.28        0.27        0.27        0.27        0.27
       wide  viscoelast        land        eat     analogu      manner       aquat   biodivers businessusu
       0.27        0.27        0.27       0.27        0.27        0.27        0.27        0.27        0.27
      emiss    freshwat        ghge  greenhous    nitrogen   terrestri    threaten       chang      impact
       0.27        0.27        0.27       0.27        0.27        0.27        0.27        0.26        0.26
       also        form       total      muscl      requir  furthermor     meatlik  manufactur      buffer
       0.26        0.26        0.26       0.26        0.26        0.26        0.26        0.26        0.26
    solubil         soy        vari     nugget       ideal         com   agreement      expand      specul
       0.26        0.25        0.25       0.25        0.25        0.25        0.25        0.25        0.25
   globular        mung
       0.25        0.25

$consum
    product    research    consumpt  substitut     percept        food       studi     barrier      altern      prefer
       0.47        0.38        0.37       0.37        0.36        0.35        0.34        0.34        0.33        0.33
     specif      market        hoek        may      method    identifi    behavior     pathway         can         way
       0.33        0.31        0.31       0.30        0.30        0.30        0.30        0.30        0.29        0.29
     target      qualit         tri     toward         one       howev        find         new     develop      impact
       0.29        0.29        0.29       0.29        0.28        0.28        0.28        0.28        0.27        0.27
    suggest       adopt  understand     rather         buy        diet       pasta       dutch      appeal    approach
       0.27        0.27        0.27       0.27        0.27        0.27        0.27        0.27        0.26        0.26
        aim       singl     attitud       mani      academ      freeli     reliabl    roininen     alterna        tive
       0.26        0.26        0.26       0.26        0.26        0.26        0.26        0.26        0.26        0.26
    acknowl         edg      tailor  wellknown  disappoint    familiar        test       sourc     willing
       0.26        0.26        0.26       0.26        0.26        0.25        0.25        0.25        0.25

$insect
       ball     oatmeal    children       food     caparro       edibl    mealworm      ashmor   childhood      dunlop
       0.57        0.57        0.54       0.47        0.46        0.45        0.44        0.42        0.42        0.42
 familiaris        gust       haidt       hood    mccauley      medium    realfood    tinguish      troduc       rozin
       0.42        0.42        0.42       0.42        0.42        0.42        0.42        0.42        0.42        0.40
     megido     willing   neophobia    respons     barbera    selfcook         may       first    recognis      chitin
       0.39        0.38        0.37       0.36        0.36        0.35        0.34        0.34        0.34        0.34
       choi    tortilla   grasshopp       cite   cockroach       covari         eli       gmuer        guth   lensvelt
       0.34        0.34        0.34       0.34        0.34        0.34        0.34        0.34        0.34        0.34
    nuessli   steenbekk         ver      hedon     disgust         tri       novel       might       studi     version
       0.34        0.34        0.34       0.33        0.33        0.33        0.33        0.32        0.31        0.30
        bit      author         fao        eat      living      visibl         dis      implic       later      second
       0.29        0.29        0.29       0.29        0.29        0.29        0.29        0.29        0.29        0.28
     explor       moder        also       rate    previous      stimuli     belgian       among    influenc      measur
       0.28        0.28        0.27       0.27        0.27        0.27        0.27        0.26        0.26        0.26
      futur     western      matric       chip    encourag     decosta     continu
       0.26        0.26        0.26       0.26        0.26        0.26        0.25
```

```
$flavor
            mask       lemonbasil           extrud            lemon           tomato            spice
            0.62             0.59             0.55             0.54             0.49             0.47
    vegetablebas          content           enhanc         confound    contentflavor              dim
            0.46             0.44             0.43             0.42             0.42             0.42
        equidist        nonlinear         smallest             spir  spirulinaextrud             tens
            0.42             0.42             0.42             0.42             0.42             0.42
           ulina            sampl         beetging             odor    chickenflavor          crablik
            0.42             0.41             0.41             0.39             0.39             0.39
        katayama           narrow             oili           oyster        oysterlik            shred
            0.39             0.39             0.39             0.39             0.39             0.39
       shredshap           shrimp            strip        stripshap           volunt           wilson
            0.39             0.39             0.39             0.39             0.39             0.39
           basil       complement cuellarbermfadez         foreseen          geosmin           glutam
            0.39             0.39             0.39             0.39             0.39             0.39
methylisoborneol              mib    milovanoviucu         propriat      spirulinarel         steepest
            0.39             0.39             0.39             0.39             0.39             0.39
           spici          chicken          control           intens           soybas          beefbon
            0.38             0.38             0.37             0.37             0.37             0.37
        unflavor            pasta           linear      mustyearthi            meati             four
            0.37             0.37             0.37             0.36             0.35             0.35
          earthi            aroma           pepper             bake            salti            shape
            0.35             0.34             0.34             0.34             0.34             0.34
          compar           season          descript           analog             test            beefi
            0.33             0.33             0.33             0.32             0.32             0.32
         beeflik            crush          fortifi         fullmeat           grassi        justright
            0.32             0.32             0.32             0.32             0.32             0.32
           modif           pastri           peanut              ppc             puff             roll
            0.32             0.32             0.32             0.32             0.32             0.32
           smoke             stuf           versus           overal          resembl           provid
            0.32             0.32             0.32             0.31             0.31             0.31
           lipid        peanutbas             term           formul           spread        substanti
            0.31             0.31             0.30             0.30             0.30             0.30
         perform            level              aim          pathway            musti              use
            0.29             0.29             0.29             0.29             0.29             0.28
           addit             beef             best             minc              fri           assess
            0.28             0.28             0.28             0.28             0.28             0.27
             tsp             base              bit         encapsul           former           liquid
            0.27             0.27             0.27             0.27             0.27             0.27
          pocket           qualit          quantit           though           method            scale
            0.27             0.27             0.27             0.27             0.26             0.26
         success            three             show          insight
            0.26             0.25             0.25             0.25
```

```
$sensori
           breslin            keast            evalu              use           bitter             test
              0.46             0.46             0.45             0.41             0.41             0.39
            assess         properti           mechan           overal            sampl            howev
              0.39             0.38             0.38             0.37             0.37             0.37
          descript           tomato            addit           compar         panelist          analysi
              0.37             0.36             0.35             0.35             0.35             0.34
            result             base         quantifi   multidimension          control           sensat
              0.34             0.34             0.34             0.34             0.33             0.33
            sensit           bakker            busch         clifford         considin          danguin
              0.33             0.33             0.33             0.33             0.33             0.33
         delahunti          heymann      kallithraka            kfchn  neurophysiolog             pend
              0.33             0.33             0.33             0.33             0.33             0.33
            precis          prolong        reitmeier             sall          septier            singh
              0.33             0.33             0.33             0.33             0.33             0.33
             thoma torrespenaranda            trice            bajec        bartoshuk           bolhui
              0.33             0.33             0.33             0.33             0.33             0.33
         capsaicin           carney          cessari       chemesthet          ciceral            duffi
              0.33             0.33             0.33             0.33             0.33             0.33
              file           hansen         hollowood             hort              iii            irrit
              0.33             0.33             0.33             0.33             0.33             0.33
              kidd           lanier npropylthiouracil          picker          pothesi            proxi
              0.33             0.33             0.33             0.33             0.33             0.33
           pungenc           sociat      swaincampbel           taster             wish           wright
              0.33             0.33             0.33             0.33             0.33             0.33
           product            agent             past         dinehart           method       understand
              0.32             0.32             0.32             0.32             0.31             0.31
              oral          percept        character           cognit          persist              cog
              0.31             0.31             0.31             0.30             0.30             0.30
            fatigu         guichard            nitiv             omit         beetging           analog
              0.30             0.30             0.30             0.30             0.30             0.29
           attribut              can         research          complex              hay         prescott
              0.29             0.29             0.29             0.29             0.29             0.29
        instrument            score           affect         interest         interact          pathway
              0.28             0.28             0.28             0.28             0.28             0.28
            combin           differ          beefbon         unflavor           liquid             reed
              0.27             0.27             0.27             0.27             0.27             0.27
              webb            studi         fullmeat             mask        physiolog       lemonbasil
              0.27             0.26             0.26             0.26             0.26             0.26
              show            limit           better          qualiti              spi             prop
              0.25             0.25             0.25             0.25             0.25             0.25

$textur
       properti   physiochem        nugget         analog            use       techniqu            ratio
           0.49         0.47          0.43           0.37           0.37           0.37             0.37
        analysi          soy    instrument          howev          optim       emulsifi           capabl
           0.36         0.35          0.35           0.35           0.35           0.35             0.35
           judg   microscopi          scan       yuliarti       structur        analogu             form
           0.35         0.35          0.35           0.35           0.34           0.34             0.33
            gum        freez         layer         gluten        develop           show            capac
           0.33         0.33          0.33           0.32           0.31           0.31             0.31
          emuls      fibrous         ideal      mouthfeel   hydrocolloid           icgn iotacarrageenan
           0.31         0.31          0.31           0.30           0.29           0.29             0.29
         analyt     creation        extrus           soup       composit           test           result
           0.29         0.29          0.29           0.29           0.29           0.28             0.28
          anoth      molecul         wheat           name      crosslink          desir             term
           0.28         0.28          0.28           0.28           0.28           0.27             0.27
         affect          tvp       generat         includ          uniqu         sausag            sampl
           0.27         0.27          0.27           0.27           0.27           0.27             0.26
         method      control    viscoelast        thicken            ppi            gel        synergist
           0.26         0.26          0.26           0.26           0.26           0.26             0.26
        process     commerci
           0.25         0.25
```

```
$accept
       vitro          eat         sourc         dont       belgium       control       sustain    singlecel
        0.49         0.47          0.46         0.45          0.43          0.41          0.41         0.41
  seafoodbas       analog         sampl        studi       influenc        compar         found       specul
        0.41         0.40          0.39         0.39          0.39          0.37          0.37         0.37
     particip      meatbas          know        grasp         salad       student           may        score
        0.35         0.35          0.35         0.35          0.35          0.35          0.34         0.34
     determin         four       without       overal        manner       product          show         food
        0.34         0.34          0.34         0.33          0.33          0.32          0.32         0.32
        choic        meati        result         term         scale        extent         adult      surpris
        0.32         0.32          0.31         0.31          0.31          0.31          0.31         0.30
      contain     fullmeat      although    agreement       analysi         hedon       perform       provid
        0.30         0.30          0.30         0.30          0.29          0.29          0.29         0.29
       enhanc        smoke       beefbon     unflavor       barrier        liquid      research      concept
        0.29         0.29          0.29         0.29          0.29          0.29          0.29         0.29
        motiv        older      competit       eventu  healthpromot  industryscal          kilo      lowcost
        0.29         0.29          0.29         0.29          0.29          0.29          0.29         0.29
          owe singlecellbas     slaughter    transport      vitrobas          test         addit        adequ
        0.29         0.29          0.29         0.29          0.29          0.28          0.28         0.28
        level      conduct  vegetablebas  environment         price           can      colleagu      suggest
        0.28         0.28          0.28         0.28          0.28          0.27          0.27         0.27
       altern        spici         agent          use        reveal        affect       explain        spice
        0.27         0.27          0.27         0.26          0.26          0.26          0.26         0.26
     descript   unfamiliar      previous       visibl       respond
        0.26         0.26          0.26         0.26          0.25

$increas
        decreas          concentr               mrp        carotenoid sharimaabdullah            appear          chickpea
           0.46              0.44              0.44              0.39            0.39              0.38              0.38
           fact             flour               due            lowest           addit             world             chang
           0.34              0.33              0.32              0.32            0.29              0.29              0.28
         import             score               tvp      flavorenhanc        hydrolyz             oppos             moist
           0.27              0.27              0.26              0.26            0.26              0.26              0.26
            led       colorimetr              erti              mrps       placement            broken             creas
           0.26              0.26              0.26              0.26            0.26              0.26              0.26
         melton              oliv           polymer               sds          solubi           solvent           stanley
           0.26              0.26              0.26              0.26            0.26              0.26              0.26
         teract         twocompon       watersolubl            fabric          hirata            intact            altern
           0.26              0.26              0.26              0.26            0.26              0.26              0.25

$like
            pasta           pathway          beetging           credenc              leav          outstrip
             0.61              0.47              0.42              0.42              0.42              0.42
            sound            tomato        lemonbasil          conceptu       mustyearthi           ceptual
             0.42              0.41              0.41              0.40              0.38              0.38
           compar         experienc            overal              cata               met             basil
             0.33              0.32              0.31              0.30              0.30              0.30
       complement cuellarbermfadez          foreseen           geosmin            glutam methylisoborneol
             0.30              0.30              0.30              0.30              0.30              0.30
              mib      milovanoviucu          propriat       spirulinarel          steepest               tsp
             0.30              0.30              0.30              0.30              0.30              0.29
            negat            higher              mask             bland        netherland             femal
             0.29              0.27              0.27              0.27              0.27              0.27
            stand
             0.25
```

$plantbas

composit	impl	makeup	satisfactori	smallscal	struc	vestment	nutrit
0.49	0.46	0.46	0.46	0.46	0.46	0.46	0.43
freez	nugget	techniqu	develop	imposs	diversif	pietsch	prevail
0.43	0.42	0.41	0.40	0.38	0.37	0.37	0.37
rear	round	sur	surround	unsus	mentat	ppn	substitut
0.37	0.37	0.37	0.37	0.37	0.37	0.37	0.36
process	structur	mimic	animalbas	generat	altern	regular	uniqu
0.34	0.34	0.34	0.33	0.32	0.32	0.32	0.32
necessarili	home	product	littl	issu	meatlik	analogu	studi
0.32	0.32	0.31	0.31	0.31	0.31	0.30	0.29
recent	date	ingredi	strategi	natur	potenti	constraint	rais
0.29	0.29	0.28	0.27	0.27	0.27	0.27	0.27
use	vari	anim	health	everi	insectsbas	stigma	resourc
0.26	0.26	0.26	0.26	0.26	0.26	0.26	0.26
envisag	face	terest	uni	vers	dekker	xiong	amin
0.26	0.26	0.26	0.26	0.26	0.26	0.26	0.26
barzegar	berman	carcinogen	cheng	dioxid	excess	jiang	kamankesh
0.26	0.26	0.26	0.26	0.26	0.26	0.26	0.26
khan	kuhnl	nadeem	predispos	preserv	rahman	sahar	thylcellulos
0.26	0.26	0.26	0.26	0.26	0.26	0.26	0.26
titanium	truli	characterist	includ	conduct	specif	asia	tainabl
0.26	0.26	0.25	0.25	0.25	0.25	0.25	0.25
rheolog							
0.25							

$expect

assimil	deliza	macfi	actual	discrep	predict
0.67	0.67	0.67	0.63	0.59	0.57
dispar	distast	gativ	kehkfnen	kole	latitud
0.56	0.56	0.56	0.56	0.56	0.56
mojet	sawyer	schifferstein	sect	sensorylik	wors
0.56	0.56	0.56	0.56	0.56	0.56
disconfirm	brain	frog	cardello	contrast	experi
0.55	0.55	0.55	0.51	0.43	0.43
hypothet	pectat	unclear	pasta	chines	magnif
0.43	0.43	0.43	0.40	0.39	0.39
assimila	chil	chocol	contradict	dren	feedback
0.39	0.39	0.39	0.39	0.39	0.39
posur	pothet	spons	switch	tioncontrast	upward
0.39	0.39	0.39	0.39	0.39	0.39
pathway	expectationexperi	lamb	adjust	neo	phobia
0.38	0.37	0.36	0.35	0.35	0.35
disappoint	familiar	credenc	leav	outstrip	sound
0.35	0.33	0.33	0.33	0.33	0.33
presum	effect	thus	perform	german	now
0.32	0.31	0.31	0.31	0.31	0.31
novel	crosscultur	food	reject	tan	ceptual
0.30	0.30	0.29	0.29	0.29	0.29
lead	predictor	gap	fischer	stieger	small
0.28	0.28	0.28	0.28	0.28	0.27
model	palat	enc	tuorila	postul	irrespect
0.27	0.27	0.27	0.27	0.27	0.27
somewhat	trast	obvious	conceptu	met	dutch
0.27	0.27	0.27	0.27	0.27	0.26
resist	via				
0.25	0.25				

```
$pea
    starchi       lupin       grain     chamber       broth        bott        defi   mushroomi      nition
       0.54        0.54        0.53        0.53        0.53        0.52        0.51        0.51        0.51
        ope        ubol        vara    varaubol     astring     powderi       dusti       green     definit
       0.51        0.51        0.51        0.51        0.50        0.50        0.49        0.45        0.44
      beani         pod         nut      earthi       fvalu        resp     spectiv    attribut      crimin
       0.42        0.41        0.40        0.38        0.38        0.38        0.38        0.37        0.37
     tribut       attri        bute      almond  newmankeul       solut      bitter        puls   australia
       0.36        0.36        0.36        0.36        0.36        0.35        0.34        0.33        0.32
     canada cholesterol      fortif       manag      repair     satieti   sportmind         usa       musti
       0.32        0.32        0.32        0.32        0.32        0.32        0.32        0.32        0.32
    describ    protocol       block    discrimin       among      phenol     display       devel   dimension
       0.31        0.31        0.31        0.30        0.29        0.29        0.29        0.29        0.29
      multi    panelist     classic     consist       degre      launch         six        alum    granular
       0.29        0.28        0.28        0.27        0.27        0.27        0.27        0.27        0.27
   threeway       nutti  manufactur         hay      ambigu       arcot      brothi  chandrahio       drake
       0.27        0.26        0.26        0.26        0.26        0.26        0.26        0.26        0.26
      ejszo  kaczmarska      litera lopetcharat  malcolmson        pars         ska        soil szymkiewicz
       0.26        0.26        0.26        0.26        0.26        0.26        0.26        0.26        0.26
  troszyucu       woodi         wou        sour
       0.26        0.26        0.26        0.25

$spirulina
        lemonbasil         mustyearthi                mask               pasta               lemon              though
              0.64                0.61                0.54                0.53                0.49                0.48
spirulinasoyextrud              promis             biomass           beetging             content              extrud
              0.48                0.47                0.47                0.44                0.43                0.43
            tomato             peratur                 tem               grahl                fill               futur
              0.43                0.42                0.42                0.42                0.42                0.39
              turn                step          expenditur                ject              someth               stood
              0.39                0.39                0.39                0.39                0.39                0.39
             usag                basil          complement   cuellarbermfadez            foreseen             geosmin
              0.39                0.39                0.39                0.39                0.39                0.39
            glutam   methylisoborneol                 mib       milovanoviucu            propriat         spirulinarel
              0.39                0.39                0.39                0.39                0.39                0.39
          steepest                 bao             foundat             intensif                 ren               zheng
              0.39                0.39                0.39                0.39                0.39                0.39
              take           microalga                even                part             pathway                firm
              0.38                0.37                0.37                0.36                0.36                0.34
            earthi              intens                serv               musti            confound       contentflavor
              0.34                0.33                0.33                0.33                0.33                0.33
               dim            equidist           nonlinear            smallest                spir     spirulinaextrud
              0.33                0.33                0.33                0.33                0.33                0.33
              tens               ulina               input             justifi          netherland             insight
              0.33                0.33                0.32                0.32                0.32                0.30
        palanisami             account               still                gain            investig                goal
              0.29                0.29                0.29                0.29                0.28                0.28
         standalon               share                deep               screw               speed               entir
              0.28                0.28                0.28                0.28                0.28                0.28
              soon                 sus          preprocess                need                left            conceptu
              0.28                0.28                0.28                0.27                0.27                0.27
              odor               small               energi              accord            experienc               impli
              0.26                0.26                0.26                0.26                0.26                0.26
               key              adjust            pronounc              acolor                alga             foveral
              0.26                0.26                0.26                0.26                0.26                0.26
         influenti              omusti         beheshtipour             central                 clt        flavorflavor
              0.26                0.26                0.26                0.26                0.26                0.26
             franc               gould       khosravidarani               learn              leitch              mobini
              0.26                0.26                0.26                0.26                0.26                0.26
       mortazavian          sohrabvandi              yeoman                mere
              0.26                0.26                0.26                0.26
```

```
$posit
       rate    familiar     present    contrast        meal       famil      iariti       intro 
       0.41        0.39        0.39        0.39        0.36        0.36        0.36        0.36 
       posi        rule         may     product       ethic multisensori     apprais      pectat 
       0.36        0.36        0.35        0.34        0.33        0.32        0.32        0.32 
        wtt    particip     context        rich       negat    individu      correl      ground 
       0.32        0.31        0.31        0.31        0.29        0.29        0.28        0.28 
     expens      actual     assimil      deliza       macfi     perceiv        food       novel 
       0.28        0.28        0.28        0.28        0.28        0.27        0.27        0.27 
       corr       iness      tiness      dispar     distast       gativ    kehkfnen        kole 
       0.27        0.27        0.27        0.27        0.27        0.27        0.27        0.27 
    latitud       mojet      sawyer schifferstein       sect  sensorylik        wors         are 
       0.27        0.27        0.27        0.27        0.27        0.27        0.27        0.27 
    gembaro       provi       these      verain     cracker       shake        hous  disconfirm 
       0.27        0.27        0.27        0.27        0.27        0.27        0.26        0.25 
       chip        late       grown        basi       decid        duce        lose    hypothet 
       0.25        0.25        0.25        0.25        0.25        0.25        0.25        0.25 
    unclear     contact         est      exceed 
       0.25        0.25        0.25        0.25 

$plant
     hydrolys        success            use        meatlik          muscl         nutrit       structur 
         0.39           0.38           0.36           0.36           0.35           0.34           0.34 
     composit        hardacr         parker           bean         improv          chand         cooker 
         0.33           0.33           0.33           0.32           0.31           0.31           0.31 
          eyr gokulakrishnan          malav          ribos        silcock         talukd      tasteless 
         0.31           0.31           0.31           0.31           0.31           0.31           0.31 
         form           loss       techniqu         select         chiang          freez           impl 
         0.30           0.30           0.29           0.29           0.29           0.29           0.29 
       makeup    satisfactori       smallscal          struc       vestment        loveday           vari 
         0.29           0.29           0.29           0.29           0.29           0.29           0.28 
          aim        develop           bone maillardreact       challeng           date        analogu 
         0.28           0.27           0.27           0.27           0.27           0.27           0.27 
          ppn      waterbind        product         materi 
         0.27           0.27           0.26           0.26 
```
